# Sexual dimorphism in bacterial infections

**DOI:** 10.1186/s13293-018-0187-5

**Published:** 2018-06-20

**Authors:** Edgar Ricardo Vázquez-Martínez, Elizabeth García-Gómez, Ignacio Camacho-Arroyo, Bertha González-Pedrajo

**Affiliations:** 1 Unidad de Investigación en Reproducción Humana, Instituto Nacional de Perinatología-Facultad de Química, Universidad Nacional Autónoma de México (UNAM), Ciudad de México, Mexico; 2 Unidad de Investigación en Reproducción Humana, Consejo Nacional de Ciencia y Tecnología (CONACyT)-Instituto Nacional de Perinatología, Ciudad de México, Mexico; 3 Departamento de Genética Molecular, Instituto de Fisiología Celular, UNAM, Ciudad Universitaria, Av. Universidad 3000, Coyoacán, 04510 Ciudad de México, Mexico

**Keywords:** Sexual dimorphism, Bacterial infection, Sex steroid hormones, Estradiol, Testosterone, Progesterone

## Abstract

**Background:**

Sex differences are important epidemiological factors that impact in the frequency and severity of infectious diseases. A clear sexual dimorphism in bacterial infections has been reported in both humans and animal models. Nevertheless, the molecular mechanisms involved in this gender bias are just starting to be elucidated. In the present article, we aim to review the available data in the literature that report bacterial infections presenting a clear sexual dimorphism, without considering behavioral and social factors.

**Main body:**

The sexual dimorphism in bacterial infections has been mainly attributed to the differential levels of sex hormones between males and females, as well as to genetic factors. In general, males are more susceptible to gastrointestinal and respiratory bacterial diseases and sepsis, while females are more susceptible to genitourinary tract bacterial infections. However, these incidences depend on the population evaluated, animal model and the bacterial species. Female protection against bacterial infections and the associated complications is assumed to be due to the pro-inflammatory effect of estradiol, while male susceptibility to those infections is associated with the testosterone-mediated immune suppression, probably via their specific receptors. Recent studies indicate that the protective effect of estradiol depends on the estrogen receptor subtype and the specific tissue compartment involved in the bacterial insult, suggesting that tissue-specific expression of particular sex steroid receptors contributes to the susceptibility to bacterial infections. Furthermore, this gender bias also depends on the effects of sex hormones on specific bacterial species. Finally, since a large number of genes related to immune functions are located on the X chromosome, X-linked mosaicism confers a highly polymorphic gene expression program that allows women to respond with a more expanded immune repertoire as compared with men.

**Conclusion:**

Notwithstanding there is increasing evidence that confirms the sexual dimorphism in certain bacterial infections and the molecular mechanisms associated, further studies are required to clarify conflicting data and to determine the role of specific hormone receptors involved in the gender bias of bacterial infections, as well as their potential as therapeutic targets.

## Background

Frequency and severity of infectious diseases clearly vary between men and women. In general, males are more susceptible to diverse bacterial illnesses than their female counterparts [1]. This sexual dimorphism is evident throughout all life and starts during the infancy stage, when male predisposition to numerous bacterial infections is evident [2]. Variations between male and female individuals in the progression and outcome of infections are intimately linked to genetic, biological, and behavioral differences, which include several factors such as the exposure to certain pathogens, sex steroid hormones, and the development of different immune responses [3–5].

In regard to genetic differences, the X chromosome contains several genes implicated in the immune response, such as genes encoding Toll-like receptors (TLR-7 and 8), cytokine receptors, transcription factors, and proteins that participate in the activity of T and B cells [6, 7]. During embryonic development, one X chromosome is randomly inactivated in females, but some genes are not silenced, providing women an immune advantage [3, 8], who present stronger and more controlled innate and adaptive immune responses than men [3, 6].

**Fig. 1:**
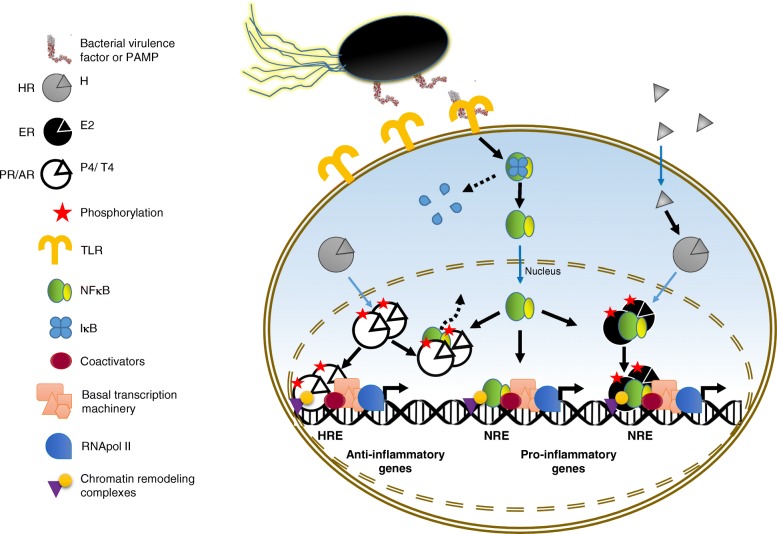
Sex hormone effects in the expression of inflammatory mediators during bacterial infections. A schematic representation of the classic mechanism of action of progesterone (P4), estradiol (E2), and testosterone (T4) (for details, see the text), as well as the cross-talk with inflammatory signaling during bacterial infections is depicted. When bacterial virulence factors or pathogen-associated molecular patterns (PAMPs) are detected through host pattern recognition receptors such as Toll-like receptors (TLRs), signaling pathways are activated, allowing dissociation of the transcription factor NFκB from its inhibitor IκB, which is subsequently phosphorylated and degraded. Active NFκB is translocated into the nucleus and binds to response elements (NREs) of target genes, allowing the expression of pro-inflammatory genes. In general, P4 induces the expression of anti-inflammatory mediators and probably downregulates the expression of pro-inflammatory factors by sequestering NFκB and inhibiting its activity (indicated by a discontinued line arrow). On the other hand, during early response to infections or sepsis, E2 and T4 display a differential inflammatory effect, in which the former induces the expression of pro-inflammatory mediators by forming a complex with NFκB, and the latter promotes an anti-inflammatory effect. During late response to infections, E2 displays an anti-inflammatory response that prevents systemic damage, while T4 shows a persistent active pro-inflammatory response that triggers systemic damage (not shown). HR steroid hormone receptor, PR progesterone receptor, ER estrogen receptor, AR androgen receptor, HRE hormone response elements

Sex hormones, besides their multiple roles in reproductive tissues, are able to influence immune cells by modulating their activity in response to infections (Fig. 1). According to the classic mechanism of progesterone (P4), estradiol (E2), and testosterone (T4) action, their cognate receptors progesterone receptor (PR), estrogen receptor (ER), and androgen receptor (AR), respectively, interact with chaperone Hsp90/co-chaperone complexes prior to their binding to hormones. When hormones diffuse into their target cell and interact with their cognate receptor, they induce a conformational change that promotes the dissociation of the chaperone/co-chaperone complex from the hormone receptor with the concomitant receptor dimerization and phosphorylation. Then, the hormone-receptor complex is translocated into the nucleus and binds to specific hormone response elements (HRE) of target genes, which allows the recruiting of co-activators, chromatin remodeling complexes, the basal transcription machinery, and the RNA polymerase II to induce gene expression [9].

Sex steroid hormone receptors have been identified in diverse cells of the immune system [3]. For example, intracellular and membrane-bound ARs have been reported in T and B lymphocytes [10], while ERs are present in macrophages, neutrophils, natural killer (NK) cells, and lymphocytes [11, 12]. PRs have also been detected in T and B lymphocytes, mast cells, eosinophils, macrophages, and dendritic cells [13, 14]. Regarding the effect of sex steroid hormones on the immune system, mediated principally by regulating the NFκB transcription factor activity, testosterone generally functions as a suppressor at early stages of infection, but as an activator during the late response to infection, as it occurs during sepsis; whereas, estradiol acts as an activator, and progesterone functions as an immunosuppressive agent, mainly during pregnancy [4, 15–17] (Fig. 1). Therefore, in response to infections, females usually activate Th2 responses and produce high levels of interleukins (IL) IL-4, IL-5, and IL-10 [14, 18–20]. On the other hand, males present predominantly Th1 responses and overproduce tumor necrosis factor α (TNF-Uα), IL-1β, IL-2, IL-6, and IL-8, which in turn are frequently associated with inappropriate outcomes such as sepsis and bacteremia [21–24]. Besides, women show both higher baseline levels and elevated production of immunoglobulins (Ig) in response to bacterial challenge than men, in particular, IgM [1, 19].

Aging and menopause also influence the immune system function, increasing the predisposition to infections [25]. During menopause, there is a significant reduction in sex steroid hormone synthesis with a concomitant decrease in immune cell levels and their functional capacity [1, 25]. Postmenopausal women and women with induced menopause due to surgical elimination of the ovaries have reduced levels of B cells and anti-inflammatory cytokines, IL-4, and interferon γ (IFNγ), while NK cell activity and levels of pro-inflammatory cytokines such as TNF-α, IL-1β, IL2, and IL-6 are increased [26–29]. These **e**ffects are partially reversed with hormone replacement therapy [25, 27], which corroborates the significant role of sex steroid hormones in the immune system function.

In agreement with the aforementioned studies, development of bacterial infections depends on the influence of gender-associated factors such as sex steroid hormones, through the modulation of differential immune responses between women and men. The aim of the present article is to review the available data in the literature about bacterial infections that present a clear sexual dimorphism, such as gastrointestinal and respiratory infections, and sepsis, among others (Table 1). Although there is increasing evidence that confirms the sexual dimorphism in certain bacterial infections and the molecular mechanisms associated, further studies are required to clarify conflicting data and to determine the role of specific sex hormone receptors in the gender bias of bacterial infections, as well as their role as possible therapeutic targets.

**Table 1:** Gender preference in bacterial infections

Type of infection	Gender preference	Main bacteria species	Associated diseases or complications	References
Gastrointestinal tract infections	Men	*Salmonella typhi*	Typhoid ileal perforation	[30]
		*Campylobacter jejuni*	Inflammatory bowel disease	[37, 38]
		*Helicobacter pylori*	Gastritis, peptic ulcer, and gastric cancer	[39]
		*Clostridium difficile*	Fulminant colitis	[47, 48]
		*Yersinia enterocolitica*	Enteritis, enterocolitis, and gastroenterocolitis	[52]
		*Vibrio spp.*	Gastroenteritis and wound infections	[57, 200, 245, 246]
	Women	Enterohemorrhagic *Escherichia coli*	Hemolytic–uremic syndrome and irritable bowel syndrome	[59, 60]
Respiratory tract infections	Men	*Streptococcus pneumoniae*	Community-acquired pneumonia	[79]
		*Mycobacterium tuberculosis*	Tuberculosis	[93–100]
		*Legionella spp.*	Legionnaires’ disease	[131, 132, 141, 133–140]
Bloodstream infections	Men	Many bacterial species	Septic shock, organ dysfunction, severe sepsis	[18, 162, 177, 163, 165–167, 173–176]
Urinary tract infections	Women	*Escherichia coli*	Acute cystitis, inflammation, and sepsis	[229, 231]
Lyme borreliosis	Women	*Borrelia burgdorferi*	Erythema migrans and facial nerve palsy	[232, 233]
Sexually transmitted infections	Women	*Chlamydia trachomatis*	Infertility, ectopic pregnancy, and pelvic inflammatory disease	[235, 237]
		*Neisseria gonorrhea*	Gonorrhea, pelvic inflammatory disease	[234]
Listeriosis	Women	*Listeria monocytogenes*	Bacteremia, meningitis	[241]
Q fever	Men	*Coxiella burnetii*	Fever, granulomatous hepatitis, myocarditis, pericarditis, and pneumonia	[243]
Wound infections	Men	*Mycobacterium marinum*	Swimming-pool granuloma or fish-tank granuloma disease	[245]

## Sexual dimorphism and bacterial infections in the gastrointestinal tract

Certain gastrointestinal infections are more recurrent and severe in men than in women. This sexual dimorphism can be explained in part because of behavioral differences during hygiene and eating practices, such as handwashing before food handling and eating, storage, refrigeration, and defrosting practices, and tendency of men to eat undercooked or raw food, as well as to ingest roadside food [30]. Although sexual dimorphism in gastrointestinal infections is mainly due to immune differences, these differences in turn can be influenced by the above mentioned behavioral dissimilarities between women and men. This is the case of typhoid fever and its complication: typhoid ileal perforation (TIP) [30]. TIP is characterized by an exacerbated inflammatory activity at Peyer’s patches, probably in response to *Salmonella typhi* exposure and re-exposure events due to behavioral factors that favors infection in men.

During gastrointestinal injury, males produce predominantly pro-inflammatory cytokines, such as IL-6 and TNF-α, as compared with females whose intestine produce anti-inflammatory factors such as IL-10 as well as protective factors of endothelial function such as a modest rise in nitric oxide (NO) levels [31]; in turn, the inflammatory response in males leads to perforation and cell necrosis at the site of infection [30]. Sex hormones signaling through their cognate receptors could play an important role in the progression of this pathology and their low incidence in women since both isoforms of ER (ER-α and ER-β) have been identified at Peyer’s patches [30, 32], and estradiol induces T cell proliferation and activity, as well as production of anti-inflammatory cytokines [32]. The differential concentrations of sex hormones between men and women influence the type of immune response that is activated. Estradiol levels are higher in women (they rise up to 1250 pmol/L during the luteal menstrual phase) than those found in men (37–210 pmol/L), and the mean threshold required to induce production of anti-inflammatory factors and to suppress production of inflammatory cytokines is 690 pmol/L; thus, an inflammatory balanced response is produced in females. On the other hand, testosterone that exhibits higher concentrations in men than in women (6.9–34.7 and 0.7–2.8 nmol/L, respectively), suppresses Th2 response and stimulates Th1 response in males, probably through the activity of ARs located in macrophages and lymphocytes that in turn regulate the differential production of cytokines, which favor the sexual dimorphism observed in this infection [33–35]. Additionally, in response to a bacterial stimulus, there is a differential expression of TLRs between females and males, which influences sexual dimorphism of gastrointestinal infections, since females show elevated levels of TLR2 and TLR4 in peritoneal macrophages and in consequence have a higher capacity to detect and eliminate pathogens than males [36].

Campylobacteriosis is another gastrointestinal infection that displays a sexual dimorphism [37]. This infection of zoonotic origin is caused by *Campylobacter jejuni* and provokes gastroenteritis, affecting predominantly men, especially young children. *C. jejuni* infections are related to the development of inflammatory bowel diseases and autoimmune pathologies such as Guillain-Barré syndrome [37, 38]. It has been proposed that this tendency is caused by behavioral, environmental, and physiological factors. Strachan and collaborators used a mouse model of infection (Myd88 adaptor protein-deficient mice, which showed persistent colonization by *C. jejuni* to favor a stable infection), under controlled laboratory conditions that were established to minimize the effects of behavior and environment. They found that bacterial colonization was present in 100% of infected male mice, in contrast to 25% of infected females. Moreover, bacterial counts recovered from feces and different tissues were higher in male than in female rodents, supporting the influence of physiological differences between sexes on the prevalence of this bacterial disease in males [37]. However, more epidemiological and experimental studies are required to clarify and/or refuse the role played by environmental and behavioral factors in sexual dimorphism of campylobacteriosis.

In addition, infections caused by *Helicobacter pylori* affect predominantly males, and its outcomes, such as gastritis, peptic ulcer, and gastric cancer, are also more frequent in males. A possible explanation for this sexual dimorphism is the protective and vigorous immune response exerted by females during *H. pylori* infections via estrogens (probably through their specific receptors) [39, 40]. In this regard, estradiol treatment in males with gastroduodenal preneoplastic alterations reverts the histological modifications such as hyperplasia and dysplasia induced by the carcinogen agent *N*-methyl-*N*′-nitro-nitrosoguanidine [41]. Moreover, male mice with gastric pathology induced by *H. pylori* infection have shown reduced expression of pro-inflammatory cytokines IFN-γ and IL-1β, increased expression levels of IL-10 and higher Th2-associated IgG1 responses after estradiol treatments. These findings have been associated with a reduction in the severity of gastric lesions caused by *H. pylori* [42].

Although cancer is not the subject of the present review, it is relevant to mention the protective effect of estrogens in order to explain their role and the ER participation during *H. pylori* infection to favor a successful outcome in females. It has been demonstrated in gastrointestinal cancer pathologies that cell proliferation pathways are regulated through ER-β. Inhibition of epithelial proliferation has been documented in a cell line of human gastric adenocarcinoma treated with 17β-estradiol [43]. Additionally, over-expression of ER-β in ER-β-lacking HCT8 cells (human colon cancer) inhibited cell proliferation by modulating cell cycle regulators such as cyclin E, and producing cell cycle arrest at G1/S phase [44]. In another study, ER-β activated by 17β-estradiol also has an inhibitory effect on colorectal cancer tumor proliferation, enhancing DNA mismatch repair by upregulating mRNA and protein levels of MLH1 [45], so a similar mechanism could also be involved in female protection against gastric cancer induced by *H. pylori* infection.

On the other hand, the expression of several *H. pylori* virulence factors is determined by other gender-specific host characteristics. Blood group antigen binding adhesin A (BabA) of *H. pylori* is a virulence factor important for bacterial binding to ABO blood group antigens expressed in the gastric epithelium and mucus layer (especially to O/Le^b^ group) and to promote the translocation of CagA oncoprotein by the type IV secretion system into the host cell cytoplasm. Recently, it has been demonstrated that *babA* expression is elevated in *H. pylori*-infected male mice as compared with lack of expression in infected females. Additionally, males showed higher bacterial colonization levels than female mice. These findings are independent of adaptive immunity or TLR signaling; thus, other gender-associated factors such as gastric physiology and differential glycosylation patterns of blood antigens can be involved [46].

*Clostridium difficile* infections include diseases ranging from antibiotic-associated diarrhea to fulminant colitis, which are often acquired in hospitals and are associated commonly with men exposed to surgery [47, 48]. *C. difficile* is characterized by forming spores that survive in the acidic environment of the stomach lumen and by producing exotoxins when germination occurs in response to exposure to bile acids in the small bowel [47]. The fact that biliary secretions that act as spore germination inductors, such as taurocholate, also known as bile salt, share the fused ring structure with steroid hormones and that both are synthesized from cholesterol has suggested that steroid hormones should also regulate the germination of bacterial spores [49]. In this regard, the effects of progesterone and progesterone analogs such as pregnenolone as well as metabolic intermediates of estrogens such as dehydroepiandrosterone (DHEA) have been analyzed on *C. difficile* spore germination. Some of these steroids inhibit spore germination (progesterone showed a half maximal effective concentration of 80.5 μM and DHEA of 168.3 μM), acting as competitive inhibitors of taurocholate, suggesting an important role of progesterone and estradiol to the resolution of infection caused by *C. difficile* [50].

A sexual dimorphism has also been reported in the disease produced by *Yersinia enterocolitica*. Yersiniosis is a zoonotic disease transmitted by domestic animals, mainly by pigs, as well as contaminated pork products or water. *Y. enterocolitica* infection provokes enteritis, enterocolitis, and gastroenterocolitis, which symptoms include bloody diarrhea, fever, abdominal pain, and vomiting; chronic cases of infection develop necrotic enteritis and purulent mesenteric lymphadenitis [51]. Yersiniosis is more recurrent in male patients of diverse ages (mainly children) than in females, and this tendency has been reported in an epidemiological study performed in Germany [52]. To our knowledge, there are no studies about the gender-associated molecular mechanism involved in the course of infection by *Y. enterocolitica*; nevertheless, there are higher serum levels of IgG4 antibodies in male patients as compared with female patients in response to *Yersinia* outer membrane proteins (Yops) [53], which are plasmid-encoded virulence factors secreted by a type III secretion system [51]; however, researchers did not provide an explanation to this result [53]. In the same regard, it has been reported that high levels of IgG4 are related with chronic exposure to antigen [54], which in turn is associated to an excessive activation of anti-inflammatory mechanisms that can be related with a resistance to treatment in males as it has been observed in other infections [55, 56]. Thus, IgG4 levels in males during *Y. enterocolitica* infections can be associated with an anti-inflammatory response that interferes with the disease outcome.

*Vibrio spp.* are marine and estuarine bacteria that cause infections from gastroenteritis to wound infections, also with a higher prevalence in males. The gastric infections by *Vibrio spp.* are associated with seafood consumption, principally raw oyster ingestion. *Vibrio* species that commonly causes gastroenteritis are *Vibrio parahaemolyticus*. *V. cholerae*, *V. hollisae*, *V. mimicus*, and *V. fluvialis*, infections with a reported male-female incidence ratio of 1.7:1 [57]. *V. cholerae* is the most representative species in the genus, with the highest proportion of cases of *Vibrio*-associated gastroenteritis (71%), their toxigenic strains belonging to O-group (mainly serogroups O1 and O139) are the cause of epidemic cholera, with 2.86 million cases and 95,000 deaths annually [57, 58]. In spite of the evident male prevalence, the molecular mechanism involved in the gender bias in *Vibrio* infections, particularly the role played by sexual hormones, has not been studied.

In contrast to the aforementioned gastrointestinal infections, enterohemorrhagic *Escherichia coli* (EHEC) O157 infections are more frequent in female individuals, with 61% of the adult cases from 2000 to 2009 in the Japanese population [59]. In another study performed in an Asian population, comparable prevalence rates for females were found since 54.3% cases in Korea and 53.7% in Japan were women [60]. A similar pattern was observed during an outbreak of enteroaggregative Shiga-toxin *E. coli* O104:H4 in Germany in 2011, where most of the infected people were women, corresponding to 58% of infected adults; furthermore, the frequency of women who developed the concomitant hemolytic–uremic syndrome was greater than men [61]. Sequels of these infections are also more frequent in female than in male patients, such as irritable bowel syndrome (IBS), a chronic disorder that causes symptoms as cramping, pain, bloating, flatulence, nausea, diarrhea, and constipation [62, 63]. In turn, aggravation of IB is associated with alterations of gastrointestinal physiology due to varying levels of sex steroid hormones throughout the menstrual cycle and to the activity of these hormones through their cognate receptors located in the gastrointestinal tract [64]. For example, low estradiol during menstruation causes intestinal inflammation by inducing mast cell degranulation and prostaglandin production that in turn are associated with visceral sensitivity and could exacerbate the response to infections [64, 65].

In general, sex dimorphism in gastrointestinal infections tends to favor a successful outcome in women, through the actions of estradiol via its intracellular receptors, which, as in other infections, regulate immune system function as well as host non-immune factors and bacterial physiology. In contrast, some infections, such as that caused by EHEC are affected by cyclic changes in female hormone levels, provoking worse outcomes in women. However, differential regulation of immune system in response to specific concentration of female hormones can be an advantage in the treatment of gastric infections.

## Bacterial infections in the respiratory tract

In general, males are more susceptible than females to lung diseases such as neonatal respiratory distress syndrome, pulmonary arterial hypertension, idiopathic pulmonary fibrosis, and asthma, and this is also observed for community-acquired and nosocomial bacterial pneumonia, in which severity and higher risk for mortality are associated with male patients [66–69]. In fact, it has been suggested that male sex is a frequent risk factor and a predictor of worse outcome for most of the respiratory tract infections, with exception of some upper respiratory tract infections (sinusitis, tonsillitis, and otitis externa) that are more frequently found in females [67]. As in other infections, the sexual dimorphism in respiratory infections has also been attributed, independently of sex hormones and genetic factors, to socioeconomic, lifestyle, behavioral, and other factors [70–72] that will not be considered in the present review. In women, these differences have been mainly attributed to a gender inequality in work and access to healthcare system [67]. Interestingly, it has been reported that there are no significant differences between the immune response of men and women athletes that could be associated with respiratory tract infection incidence, suggesting that in this population there is no sexual dimorphism [73].

It has been reported that biological factors could also contribute to the sexual dimorphism observed in respiratory tract infections, such as anatomical and physiological differences in the respiratory tract [67]. In women, the higher prevalence of rhinosinusitis could be due to the fact that they have smaller ostia than men, which is more frequently associated with obstruction and therefore with infection [74]. In contrast, it has been suggested that the higher prevalence of lower respiratory tract infections in boys during the early years of life is due to peripheral airway width disproportions that are more marked in males [75].

The effects of sexual hormones on the immune response of the respiratory tract depend on the experimental model. It has been demonstrated that estradiol induces an increase in inflammatory cytokines (such as TNF-α and IL-6) and chemokines (such as monocyte chemoattractant protein 1 and macrophage inhibitory protein 1α) in the lungs of ovariectomized mice infected with *Pseudomonas aeruginosa*, which in turn is associated with a neutrophil dysfunction and a worst outcome as compared with vehicle-treated female mice and intact males. Interestingly, this effect was ER dependent [76]. In contrast, in an experimental model of pleurisy (pleural inflammation) in ovariectomized rats, estradiol treatment prior to the inflammatory insult was associated with a decrease in NO production, migration of polymorphonuclear cells, and tissue injury, which was also ER dependent [77]. Furthermore, it has been suggested that estradiol promotes an increase of IgA transport into the mucosa of the respiratory tract protecting against pneumonia [78]. More studies are required to elucidate the beneficial or detrimental effects of estradiol and other sexual hormones during respiratory tract inflammation.

The incidence of community-acquired pneumonia is higher in men than in women, and this incidence increases by age, especially in infections with *Streptococcus pneumoniae*, *Streptococcus pyogenes*, *Chlamydophila pneumonia*, and *Legionella pneumophila* [79–86]. On the other hand, a gender preference for pneumonia caused by drug-resistant pathogens such as *P. aeruginosa*, extended-spectrum-beta-lactamase-positive *Enterobacteriaceae*, and methicillin-resistant *Staphylococcus aureus* has not been established, as this gender preference depends on the studied population [87–89]*.* Females have a 57% reduced risk to acquire ventilator-associated pneumonia after mechanical ventilation, which has been reported to affect 72.7% of males, mainly due to Gram-negative bacterial infections [90, 91]. In contrast, women under the age of 50 years are more susceptible to respiratory infections by *Mycoplasma pneumoniae* [92]. For a comprehensive and complete review of the incidence and severity in respiratory tract infections refer to [67].

Tuberculosis, caused by *Mycobacterium tuberculosis*, is one of the most studied models regarding sexual dimorphism in respiratory tract infections. In humans, tuberculosis is one of the leading causes of death from infectious diseases. It was diagnosed in about 8.6 million people in 2012, and it has been predicted that one third of the population in the world has a latent infection [93]. Some of the risk factors for tuberculosis infection are malnutrition, smoking, diabetes, and sex [94]. Although the prevalence of tuberculosis in males depends on the geographic region, there is a general trend for the male-to-female ratio affecting more males with poorer outcome in developing countries, including a higher risk of mortality [93, 95–100]. This male gender prevalence is also observed when combining other risk factors such as human immunodeficiency virus (HIV) infection, diabetes mellitus, and smoking [101–104]. It has been reported that Bacillus Calmette-Guerin (BCG) unvaccinated males display a stronger IFN-γ response against the tuberculin purified protein derivative (PPD) than females, which suggests that males display a stronger immune response that could be associated with an uncontrolled inflammatory response and a poor prognosis during *M. tuberculosis* infection [105]. During tuberculosis infection, there is also a differential immune response characterized by females showing higher levels of the C-X-C Motif Chemokine Ligand 9 (CXCL9) and males showing higher levels of the Platelet Derived Growth Factor Subunit B (PDGFB), serum C-reactive protein, and specific antibodies against *M. tuberculosis* than their gender counterpart, highlighting a stronger innate and humoral immune response in males [106, 107]. Furthermore, the plasmatic levels of the anti-tuberculosis drugs, isoniazid, and pyrazinamide are lower in adult males than in females. This could be related to the worst outcome in the treated males [108]. Interestingly, this higher proportion of affected males is not observed in children or young adolescents, suggesting the participation of sex hormones in the pathogenesis of tuberculosis [109, 110]. In line with this, it has been reported that the prevalence of tuberculosis associated deaths in a mentally retarded population was lower in medically castrated males (8.1%) compared with intact males (20.6%) and females (15.8%) [111]. Women who underwent medical oophorectomy present an increased risk of mortality because of tuberculosis [112]. However, it has been also demonstrated that this male bias to tuberculosis infection is due to genetic factors, illustrated by X-linked TLR8 gene polymorphisms that have been associated with increased susceptibility to tuberculosis in male children [113], as well as by the X-linked Mendelian susceptibility to mycobacterial diseases due to mutations in IKBKG and CYBB genes, that participate in the induction of IL-12 by mononuclear cells and the activation of the respiratory burst in macrophages, respectively [114]. Besides, it has been reported that elderly women with decreased estrogen levels (postmenopausal women) have an increased risk of chronic lung infections produced by nontuberculous *Mycobacterium* infections [115]. Particularly, it has been found that decreased DHEA (an endogenous intermediate in the conversion of cholesterol to estrogens and androgens) levels in elderly women are associated with *M. avium* complex infection, a nontuberculous mycobacterial lung infection [116]. These studies suggest that nontuberculous *Mycobacterium* infections are also influenced by hormones, which in turn explains the sexual dimorphism in overall *Mycobacterium* infections in the respiratory tract.

Animal models have been used to further investigate the role of sex hormones in the pathophysiology of tuberculosis. Early studies demonstrated that male mice are more susceptible than female mice to infection with *M. intracellulare* and *M. marinum* and that testosterone treatment increases female and castrated male mice susceptibility to *M. marinum* showing more severe granulomatous lung lesions, contrary to the protective effect observed after estradiol treatment in ovariectomized mice exposed to *M. avium* [117–119]. Sex bias has also been reported in *M. tuberculosis* mice infection, where male mice are at greater risk of mortality and show higher numbers of bacilli burdens in lungs with a lower pro-inflammatory response (constituted mainly by decreased levels of lymphocytes, macrophages, and expression of pro-inflammatory cytokines) than female mice and castrated mice [120]. Interestingly, it has been demonstrated that medroxyprogesterone acetate (DMPA), a progestin commonly used as a contraceptive, decreases the cytokine response to *M. tuberculosis* in C57BL/6 and BALB/c mice [121].

Regarding other animal models, it has been reported that bovine tuberculosis caused by *M. bovis* also displays a worse outcome, higher mortality rate, and more rapid progression rates in males than in females, and this difference has been attributed to an immunosuppression induced by testosterone in males [122–124]. The above studies clearly demonstrate a sexual dimorphism in *Mycobacterium spp.* respiratory tract infections affecting more males than females, which is influenced by estradiol and testosterone and the genetic background. The participation of sex hormone receptors in this dimorphism has not been elucidated.

In the natural model of murine respiratory mycoplasmosis induced by *M. pulmonis*, it has been demonstrated that male mice display a more severe clinical outcome than females, with higher rates of mortality and worse signs of alveolar disease, but in contrast to other mycoplasmosis, the removal of gonads decreases the severity of lung injury and bacilli number in both sexes [125]. Interestingly, in this study, sex differences in *M. pulmonis* infection were remarkable since male mice showed an acute alveolar inflammatory response (edema, hemorrhage, and neutrophil infiltrate) whereas female mice showed a chronic peribronchial inflammatory response (macrophage and few neutrophil infiltrates), which explains the worse outcome in males [125].

Legionnaires’ disease is a severe form of infection with *Legionella* species, which are Gram-negative bacteria that are ubiquitously found in natural and artificial aquatic habitats, as well as in moist soil and mud [126, 127]. Legionellosis is mainly characterized by pneumonia with a high incidence of mortality rates, and although it is frequently observed as sporadic cases, it has been also reported in community and hospital outbreaks mainly due to contamination in the air conditioning systems [128–130]. It has been reported a strong association of legionellosis with male gender in many European, American, and Asian countries, as well as in Australia, and in fact, male gender is considered as a poor prognostic factor [131–141].

In contrast to the mentioned respiratory tract infections, there are bacterial infections that affect more females than males. These gender differences have been clearly demonstrated in cystic fibrosis disease (CF) that is characterized by a dysregulated inflammatory response and an altered cytokine profile in the lungs that is associated with a higher prevalence of bacterial infections (mainly due to *P. aeruginosa*) and poorer lung and respiratory muscle function in females than in males [142–144]. Differential estradiol levels between females and males have been proposed as a factor that explains the sexual dimorphism in CF-associated infections because differences in gender lung function are observed after puberty and are correlated with estrogen levels during the menstrual cycle [145]. In particular, high estradiol levels in CF females have been associated with the conversion of *P. aeruginosa* in a highly pathogenic mucoid bacteria that induces parenchymal damage in the lung [146]. Moreover, the increase in estradiol levels in CF females upregulates the expression of the secretory leucoprotease inhibitor via ER-β, that in turn inhibits the TLR-dependent IL-8 release in CF bronchial epithelial cells predisposing to *P. aeruginosa* infection and colonization [147]. It has been demonstrated that *P. aeruginosa* pulmonary infection is also associated with a worse outcome in female mice, which is characterized by a more pronounced weight loss, higher numbers in bacterial load, and a higher inflammatory response than those observed in male mice [148]. It has been also reported that female sex is associated with an increase in the mortality rate in a model of CF mice with *P. aeruginosa* pulmonary infection [149]. Furthermore, estradiol administration increases the severity of *P. aeruginosa* pneumonia in adult CF male mice by upregulating IL-17 signaling and promoting lung tissue damage [150].

Interestingly, it has been reported that perinatal pulmonary infection induced by the intracellular bacteria *Chlamydia muridarum* displays a sexual dimorphism in terms of hippocampal expression of corticosteroid receptors associated with an altered stress response that emerges during adulthood, in which females are more affected by neonatal infection than males as demonstrated by an increase in glucocorticoid receptor and tyrosine hydroxylase content in adulthood, that in turn leads to a dysregulation of the hypothalamic–pituitary–adrenal axis [151]. This highlights the importance of the sexual dimorphism in bacterial infections not only in the affected tissue but also in other organs such as the brain.

There are epidemiological studies reporting that air pollution confers a higher susceptibility to respiratory tract diseases to females as compared with males [152–154]. Although there are some controversies about this prevalence in humans, studies in mice have demonstrated that after ozone-induced oxidative stress, there is a decrease in survival rate that is more pronounced in female than in male mice in *Klebsiella pneumoniae* lung infection, which is in contrast to the lower survival rate observed in male mice in ozone-free conditions. These differences have been associated with a compromised spleen, reduction in functional activity of surfactant protein A, increased plasma cortisol levels, and reduced phagocytic ability of alveolar macrophages in ozone-exposed female mice [155–157]. Ozone exposure alone is associated with an increased inflammatory response, higher lung vascular permeability, and higher number of polymorphonuclear neutrophils in bronchoalveolar lavage in female mice [158]. Interestingly, it has been proposed that female gonadal hormones (estradiol and progesterone) are protective against *K. pneumoniae* pulmonary infection; however, the protective effect is lost after ozone exposure, and even leads to a worse outcome that could be related to the excessive activation of the inflammatory pathway in response to estradiol and ozone exposure [159].

Although there is an evident sexual dimorphism in both humans and animal models of respiratory tract infections, the participation of hormone receptors has been poorly explored. The fact that estradiol and testosterone have been proposed to participate in the pathogenesis of the mentioned respiratory tract infections strongly suggests that intracellular and/or membrane hormone receptors are involved in this sexual dimorphism. One example is the participation of ER-β in the inhibition of TLR dependent IL-8 release in CF bronchial epithelial cells in the model of CF mice infected with *P. aeruginosa*, which clearly demonstrates an important role of hormone receptors in the gender bias of *P. aeruginosa* induced pneumonia [147]. Therefore, further studies are required to elucidate the participation of hormone receptors in the sexual dimorphism observed in bacterial infections in the respiratory tract that in turn could help in prognosis and management.

## Sexual dimorphism and bacterial sepsis

### Sepsis

The host response against a bacterial infection that leads to a systemic inflammatory response syndrome is defined as sepsis, which is characterized by the increase in TNF-α, IL-1β, IL-6, and IL-8 cytokine secretion mainly by macrophages after the recognition of bacteria or bacterial-derived lipopolysaccharide (LPS) via TLRs [21, 160]. Although there are some controversies about the sex-specific response in sepsis patients [161], it has been stated that gender plays a significant role in post-injury pathogenesis and its association with the susceptibility to sepsis by affecting more males than females [162, 163]. This gender bias is explained only in part because of occupational, hygiene, and sport activities differences among men and women [164]. In fact, male gender is considered as one of the four risk factors associated with the development of sepsis in general surgical and post-traumatic patients, with a hospital mortality rate of 70% in male and 26% in female patients [18, 165, 166]. Moreover, it has been reported that male gender along with HIV infection is a risk factor for bacteremia, suggesting an important role of immune suppression [167]. The pathological response to trauma-hemorrhage in rodent males, ovariectomized females, and testosterone-treated females is characterized by a severe suppression of the immune response and multiple organ dysfunctions, leading to a high incidence of sepsis [168–170]. Interestingly, these effects are not observed in proestrus rats, estradiol-treated ovariectomized rodents, castrated male rodents, and estrogen-treated males, which clearly point out the participation of estradiol and testosterone in the sex bias of sepsis after trauma-hemorrhage [168, 171, 172].

Moreover, a differential immune response between genders during sepsis has been observed and is characterized by a specific profile of pro- and anti-inflammatory cytokines. In particular, male patients display higher levels of pro-inflammatory cytokines such as IL-6 and TNF-α as well as increased levels of bacteremia markers like procalcitonin in sepsis than females [173–176]. In contrast, female patients display higher levels of the anti-inflammatory cytokine IL-10 than males [18]. These differences between immune responses before and after trauma-hemorrhage leading to sepsis suggest that females display an early and controlled pro-inflammatory response that prevents infection which is followed by an anti-inflammatory response that avoids systemic damage, while males display a late and persistent active pro-inflammatory response leading to systemic damage. Besides, the fact that sexual dimorphism in severe sepsis is also observed in prepubertal children and elderly people suggests that genetic and environmental factors are also involved in this gender bias [177–179].

### Experimental sepsis

Animal models have been widely used to study sex differences in regard to immune response and clinical effects of sepsis. In particular, cecal ligation and puncture (CLP) in mice, leading to polymicrobial sepsis, has demonstrated that proestrus female mice display a higher survival rate than male mice, along with a marked increase in splenocyte proliferation and IL-2/IL-3 release as compared with male mice, suggesting that immune homeostasis regulation is better regulated in females than in males [180]. Remarkably, beta-glucan therapy promotes survival in mice subjected to CLP and displays a better protective effect in female than in male mice by enhancing bacterial clearance, which is dependent on sex hormones [181]. The use of estradiol or DHEA in male mice subjected to CLP improves splenocyte, macrophage, and T cell activity [182, 183]. Sex differences favoring female mice after CLP are similar to the better prognosis observed in female patients undergoing major abdominal surgery; in these cases, male patients present decreased numbers of circulating B and T lymphocytes and NK cells after abdominal surgical intervention [184]. It has been proposed that sex differences in prognosis after abdominal trauma and subsequent sepsis are due to an enhanced and strictly regulated antimicrobial host defense in the female peritoneum as compared with that of males, which in females, is characterized by a higher number of quiescent leukocytes (mainly T and B lymphocytes), competent resident macrophages (that highly express TLRs and display elevated phagocytosis), and immunomodulatory CD4 lymphocytes [11, 36]. Furthermore, estradiol and progesterone are necessary to maintain homeostasis regulation of resident leukocytes in the female peritoneum, while testosterone disrupts the trigger of an appropriate innate immune response against polymicrobial insult [36, 185].

Another sepsis model in animals is the “two-hit” model that consists in trauma-hemorrhage-induced shock (first hit) and subsequent sepsis (second hit), in which females display a better outcome and survival than males, presenting lower plasma levels of pro-inflammatory markers (IL-6, TNF-α, and prostaglandin E(2)) after sepsis [186]. However, a recent study failed to find a correlation between inflammatory markers and the different outcome observed in female and male mice in the “two-hit” model [187]. A protective effect has been observed in proestrus rat during hemorrhagic shock as compared with other estrous cycle phases; this has been attributed to the highest levels of estradiol in proestrus phase [188]. Protective effects of estradiol against sepsis following hemorrhagic shock have been reported in trauma-hemorrhage-induced lung and hepatic injury by decreasing the TLR4-dependent release of pro-inflammatory cytokines (IL-6, TNF-α, and macrophage inflammatory proteins 1α and 2) and chemokines (monocyte chemoattractant protein-1 and keratinocyte-derived chemokine), as well as the induction of the inducible nitric oxide synthase (iNOS) expression, neutrophil influx, and tissue damage [189–191]. On the other hand, higher levels of testosterone and its derivatives (such as 5α-dihydrotestosterone, DHT) are associated with negative effects during trauma-hemorrhage that are associated to immunosuppression and subsequent sepsis in mice. In particular, DHT has been implicated in the decrease of splenocyte proliferation; macrophage function; decline of IL-1, IL-2, IL-3, IL-6, and IFN-γ release by splenocytes and peritoneal macrophages; and a marked increase of IL-10 levels [170, 192, 193]. In addition, it has been reported that testosterone downregulates the expression of the major histocompatibility complex (MHC) class II receptor on splenic and peritoneal macrophages after trauma-hemorrhage, reinforcing immune suppression [194]. The role of testosterone in the suppression of the immune system in the sepsis model has been confirmed by using the antagonist of the AR, flutamide that in turn improves survival and organ function in male mice [195, 196].

In vitro studies have shown that after LPS exposure, TNF-α levels are lower in peripheral blood cells isolated from premenopausal women than those from men and postmenopausal women; however, this was not associated to estradiol or testosterone levels in premenopausal women [197]. In addition, it has been reported that estradiol and LPS treatments in peripheral blood mononuclear cells isolated from women and men healthy volunteers induce the secretion of TNF-α and IL-6, while estradiol and LPS treatments decrease IL-10 secretion only in male sepsis patients, which suggests that the worst prognosis in males after sepsis is associated with a non-regulated immune response [198]. More studies are required in order to elucidate the role of IL-10 in sepsis affecting males since the increased levels of this cytokine are associated with a worse outcome by exerting suppression in the immune response, whereas its reduction leads to a non-controlled pro-inflammatory response. Interestingly, both effects are dependent on sex hormones.

One of the most used models to simulate bacteremia is LPS injection into bloodstream, which is known as experimental endotoxemia. Although there are controversies about the mechanisms involved in the sexual dimorphism of LPS response, it is accepted that males are more affected than females in LPS-induced endotoxemia [199, 200]. It has been reported in mice and rat models that males and ovariectomized females show higher levels of IL-6- and LPS-binding protein (LBP, that promotes a pro-inflammatory cellular response) after LPS-induced endotoxemia [20, 201]. An increase in visfatin expression levels (an adipokine with pro-inflammatory properties) has been reported in the serum, liver, and adipose tissue from male and female rats after LPS-induced endotoxemia, and this increase is higher in the adipose tissue of ovariectomized rats that also display a worse outcome and higher levels of mortality than intact animals [202, 203]. In contrast, it has been reported that male rats display an early suppressive immune response (decreased levels of TNF-α as compared with females) after LPS injection that is testosterone dependent, confirming an increased susceptibility to sepsis in males as compared with females [204]. Furthermore, an acute pro-inflammatory response characterized by a higher increase in TNF-α levels in female than in male calves was observed after LPS treatment, which was associated to a better outcome in females [205]. In line with this, women show a higher pro-inflammatory innate immune response during experimental endotoxemia than men, as revealed by a higher leukocyte sequestration, greater levels of the pro-inflammatory cytokine TNF-α, and increased levels of the LBP without changes in the anti-inflammatory cytokine IL-10 [206]. Further studies are required to overcome controversies in the elucidation of the molecular differences involved in gender bias after LPS-induced endotoxemia.

Although a marked sexual dimorphism in favor of females is observed in the development of sepsis, it has been proposed that in some cases females display a higher risk than males to severe shock once sepsis has occurred. This is the case of the superantigen toxic shock affecting more women than men, which has been associated with menstrual infections [207]. Superantigens are known as potent T cell activators that bind to the class II MHC and T cell receptors [208]. Therefore, a toxic shock is produced by the superantigen-induced T cell hyperactivation that leads to an increase of plasma pro-inflammatory cytokines (IL-1, TNF-α, TNF-β, and IFN-γ), profound hypotension, and multiorgan failure [208–210]. Despite the fact that superantigen toxic shock gender bias could be explained by its relation to menstrual-associated infections, it has also been demonstrated that female mice are more sensitive than males to superantigen-mediated toxic shock, which is associated with an ER-dependent increase in plasma TNF-α levels, liver damage, and lower survival in female mice, confirming the role of sex hormones in sexual differences of superantigen toxic shock [211]. This is also true for animal models in which the gender bias is influenced by the specific model of study and bacteria. In particular, estradiol administration prevents bacteremia in ovariectomized rats intraperitoneally inoculated with *Enterococcus faecalis* by increasing TNF-α and NO levels [212]. Male mice are more susceptible to *Streptococcus pneumoniae* intravenous administration than females by showing higher mortality rate, weight loss, and body temperature decrease, along with an increase in blood pro-inflammatory cytokines such as IL-6, IL-17A, RANTES, and IFN-γ as compared with female mice [81]. On the other hand, *Listeria monocytogenes* systemic infection is more prevalent in female mice due to the estrogen downregulation of IFN-γ and IL-10 induction that leads to an immune suppression [213].

### Genetic-dependent effects

Interestingly, it has been demonstrated that female X chromosome mosaicism (due to X random inactivation) provides an expanded repertoire of immune functions in females as compared with males [214]. Since a large number of genes related to immune functions are located on the X chromosome, X-linked mosaicism confers a highly polymorphic gene expression program that is specific to different cell subpopulations which respond differently to particular immune challenges [215]. It has been reported that mosaic mice with a heterozygous deficiency of the X-linked Nox2 gene (that encodes the catalytic subunit of the NADPH oxidase complex in phagocytes) displays a clear advantage during CLP as compared with wild-type animals, which is characterized by lower plasma IL-6 levels, reduced oxidative stress, higher bacterial clearance and improved survival, highlighting the contribution of genetic factors in the sexual dimorphism during sepsis [214]. This finding was further confirmed in a similar model with a heterozygous deficiency of the X-linked Irak1 gene (involved in the activation of the innate immune response) that displays better survival, lower plasma IL-6 and IL-10 levels, and non-altered splenic B and T cell populations as compared with wild-type animals in a CLP model [216]. Furthermore, it has been reported that mesenchymal stem cells obtained from female mice display a greater protective effect of myocardial function than those derived from male mice in a model of endotoxemia, confirming that genetic factors are also responsible for the gender bias in sepsis [217].

It has been suggested that a dimorphic expression of TLR4 is associated with a differential immune response between males and females [218]. In particular, higher levels of TLR4 expression in macrophages from male mice have been associated with a damaging and uncontrolled pro-inflammatory response following endotoxic shock induced by LPS administration [20]. More studies are required to demonstrate the sexual dimorphism in TLR4 expression since another study failed to find this differential expression between macrophages from male and female mice [219]. Although there is a clear prevalence of sepsis in males, sexual dimorphism does not always favor females since it depends on the population evaluated as well as on the bacterial species (Table 2). Interestingly, the differences in gender susceptibility to bacteremia among different populations highlight the importance of genetic background in the sexual dimorphism and sepsis.

**Table 2:** Gender preference in sepsis induced by specific bacterial species

Bacteria species	Gender preference	Population	References
*Staphylococcus aureus*	Women	Israel	[250]
	Men	United States of America	[251]
*Staphylococcus aureus* (methicillin resistant)	Women	Brazil	[252]
	No differences	United Kingdom	[253]
	Women	United States of America (hemodialysis patients)	[254]
	Men	Australia, Sweden, Denmark, Germany, Spain, United Kingdom, United States of America	[255–258]
*Salmonella enterica* (non-typhoidal)	Men	Six regions in Finland, Australia, Denmark, and Canada	[259]
*Escherichia coli* (extended-spectrum β-lactamase-producing)	Women	Cancer patients	[260]
*Escherichia coli*	Women	Denmark	[261]
*Klebsiella spp.*	Men	Denmark	[262]
*Streptococcus milleri*	Men	Canada	[263]
*Streptococcus spp.*	Men	United States of America	[251]
*Aeromonas spp.*	Men	Spain (chronic liver disease or neoplasm)	[264]

### Estrogen and androgen receptor-dependent effects

The fact that sex hormones participate in the gender bias observed in sepsis strongly suggests the participation of their receptors during this process, particularly ERs [220]. It has been demonstrated that treatment with specific ER-α and ER-β agonists, propyl pyrazole triol (PPT), and diarylpropiolnitrile (DPN), respectively, induce protective effects on intestinal microcirculation in both male and ovariectomized rats during sepsis, providing a plausible explanation of homeostasis regulation of resident leukocytes in the female peritoneum [221]. Interestingly, it has been reported that after trauma-hemorrhage, PPT inhibits the TNF-α and IL-6 release by Kupffer cells and promotes their production in splenic macrophages probably by the MAPK signaling pathway, while DPN has the same effect on alveolar macrophages and peripheral blood mononuclear cells. In addition, IL-10 secretion was decreased by PPT on Kupffer cells and by DPN on peripheral blood mononuclear cells, suggesting a differential role of ER-α and ER-β in the protective effect of estradiol after trauma-hemorrhage that also depends on the specific tissue compartment [222–224]. Furthermore, PPT administration prevents the splenic T cell suppression observed after trauma-hemorrhage by enhancing IL-2 and IFN-γ secretion via MAPK, NF-κB, and AP-1 signaling pathways, while it inhibits NF-κB and AP-1 signaling pathways on Kupffer cells to decrease iNOS expression and prevent hepatic damage [225, 226]. On the other hand, treatments with the ER-β selective agonist, WAY-202196, exert a protective effect in both male and female rodents subjected to CLP by decreasing TNF-α and IL-6 levels, reducing bacteremia, and maintaining intestinal integrity [227]. It has been recently reported that raloxifene, a selective ER modulator, reduces the effects of LPS-induced endotoxemia in ovariectomized rats via the induction of antioxidant and anti-inflammatory proteins such as heme oxygenase 1 (HO-1) and heat shock protein 70 (HSP70), respectively [203]. Further studies are required to elucidate the participation of other sex hormone receptors by using specific agonists and antagonists, such as in the case of flutamide, an AR antagonist that displays protective effects in sepsis models, as previously mentioned [228]. Moreover, the role of PRs during sepsis has not been assessed. The study of the participation of both membrane and intracellular hormone receptors would provide an extensive repertoire of pharmacological treatments to prevent or manage sepsis.

## Other bacterial diseases

Among other diseases that display a sex-based predominance are urinary tract infections (UTIs), which are present in females more often than in males [229]. UTIs in women occur more frequently than in men due to anatomical and physiological differences; for example, the urethra is shorter than that of men, and the distance between the anus and urethral meatus is also shorter in women. Additionally, the perimeatal region has less humidity in men than in women and prostatic secretions show antibacterial activity [230]; therefore, the probability of bacterial colonization and development of infection is lower in men. Seventy percent of these infections are related to *E. coli* strains. It has been reported that 65% of hospitalized elderly patients with UTI that have developed sepsis are women, similarly to the 54.8% of cases with a fatal outcome that has been documented in women [229]. Menopause is one of the risk factors of UTIs mainly due to the reduction of sex hormone levels, predominantly estrogens. When estrogen levels are low, as in postmenopausal woman, urogenital colonization of lactobacilli is decreased, which in turn produce a pH rise that allows the growth of *Enterobacteriaceae*, particularly *E. coli* [231].

Lyme borreliosis, a vector-borne inflammatory disease produced by *Borrelia burgdorferi*, is more common in women in the European population, frequently older than 44 years with recurrent infection and previously diagnosed with erythema migrans, the cutaneous manifestation of the infection. In contrast with men, women show an increased secretion of cytokines IFN-γ, IL-4, IL-6, IL-10, and TNF-α. In addition, elevated ratios of IL-10/TNFα and IL-4/IFN-γ have been observed in postmenopausal women, suggesting increased anti-inflammatory and Th2-based immune responses, while pro-inflammatory activity is reduced, which could explain the prevalence and recurrence of *B. burgdorferi* in this population [232]. Neurological form of disease, Lyme neuroborreliosis, is also common in female patients, mainly in girls, who usually present facial nerve palsy; in contrast, it is suggested that boys have a stronger immune response than girls during *B. burgdorferi* infection since boys have a higher level of cerebrospinal fluid inflammation (pleocytosis) [233]. It is suggested that during infancy males have a stronger innate immunity compared with females, presenting higher counts of basophils, monocytes, NKs, and higher levels of inflammatory mediators than females, as it has been reported after stimulation with LPS or mitogens [33]. Contradictory findings in Lyme borreliosis also can be related with differences in sex hormonal levels between women and men, children and adults, and variations through menstrual cycle and menopause, which can differentially influence the immune system function.

Sexually transmitted infections also have female predominance; as it is the case of infections of the genital tract caused by *Chlamydia trachomatis* and *Neisseria gonorrhoeae.* Susceptibility to both diseases is related to estradiol and progesterone levels, which throughout the menstrual cycle regulate the differential recruitment and function of immune cells and other components of the immune system [25, 234, 235]. An example of the influence of estrogens in *C. trachomatis* infection is observed when infected HeLa cells are treated with 17-β-estradiol or with diethylstilbesterol (a synthetic estrogen analog), enhancing the adherence, growth, and elementary body formation of this bacterium [236]. Moreover, β-estradiol levels present an inverse correlation with the cervical concentration of cytokines IL-1β, IL-6 and IL-10 during primary chlamydial infections. Progesterone also presents a negative correlation with IL-1β in women with recurrent infections [237]. High estrogen levels also influence the susceptibility of females to gonococcal infection since *N. gonorrhoeae*-infected mice treated with 17β-estradiol developed bacteremia quickly, due to the effect of estradiol on the levels and function of polymorphonuclear leukocytes, that become unable to eliminate gonococci [238]. Besides, high levels of progesterone, such as those observed during the luteal phase of the menstrual cycle, also promote gonococcal infection in human cervical epithelial cells through the increase of Akt kinase activity, which in turn increases NOS expression and NO production, favoring the *N. gonorrhoeae* survival and replication [239].

*Listeria monocytogenes* infection is a disease acquired by contaminated food ingestion; however, the infection reaches the spleen, the liver, and the bloodstream causing bacteremia and, in some cases, reaches the brain producing meningitis [240]. Listeriosis predominantly affects females, and it has been mainly attributed to increased levels of IL-10, which has been observed in infected female mice that also show elevated bacterial counts in the liver and spleen, as well as higher rates of mortality than males. IL-10 acts as an immunosuppressor, by inhibiting Th1 differentiation and synthesis of Th1 cytokines, as well as by suppressing macrophage effector function, antigen presentation, and proliferation of T cells. On the contrary, infected male mice have elevated levels of IFN-γ that helps to the resolution of disease, contributing to male-resistance to Listeriosis [241]. It has been observed that estrogen action influences female predisposition to Listeriosis by modulating the immune response to infection. When *Listeria*-infected mice are treated with β-estradiol, there is a reduction of monocytes and leukocytes at the infection site, and cells obtained from peritoneal exudates have an increased expression of IL-4, IL-10, and transforming growth factor-β (TGF-β), while IFN-γ, IL-12, and TNF-α expression is decreased, which in turn is related to a suppression of bactericidal activity and to a shift of Th1 to Th2-response [242].

On the contrary, infection produced by *Coxiella burnetii*, known as Q fever, is a disease that preponderantly affects males. Patients with Q fever present diverse symptoms such as fever, granulomatous hepatitis, myocarditis, pericarditis, and pneumonia, which occur more frequently in men. Ovariectomized mice infected with *C. burnetti* and treated with 17β-estradiol display reduced spleen bacterial load and granuloma, suggesting that sexual dimorphism in *C. burnetti* infection is linked to sex hormones action [243]. Variations in the infection with *C. burnetti* between male and female mice are associated with differential gene expression programs. For example, in male mice, an upregulation of IL-10 and IFN-γ genes has been observed, while genes that encode defensins were downregulated, increasing bacterial proliferation. On the other hand, in female mice, there are changes in the regulation of genes involved in circadian rhythm, that are related to estrogens secretion and IFN-γ production, which in turn are possibly associated with the successful control of infection [244].

Wound infections caused by marine bacteria also display male predominance, such as the case of *M. marinum*, an aquatic bacterium that produces the swimming-pool granuloma or fish-tank granuloma disease, which progresses in necrotizing lesions that in some cases reaches tendons and bones [245]. Marine bacteria of *Vibrio* genera that produce infections in skin lesions are represented by *V. vulnificus*, *V. alginolyticus*, *V. parahaemolyticus*, and non-O1 *V. cholerae*. Wound infections by *Vibrio spp.* mainly occur in men over 50 years of age with a preexisting lesion that is exposed to contaminated seawater or seafood, developing edema, cellulitis, vesicles, and necrosis [245, 246]. The low frequency of these infections in women has been associated with a protective role of estrogens against the endotoxins of *V. vulnificus* [200, 245].

Also, periodontal infections show sexual dimorphism, for instance, men are more susceptible than women to develop periodontitis, which as other infections are mainly attributed to the elevated production of pro-inflammatory cytokines in response to differential gene regulation through the action of sex steroid hormones [247]. In this respect, Cotti and others found that patients with endodontic infection (apical periodontitis) have increased levels of cytokine IL-2; however, these levels are considerably more elevated in men than in women [248]. In addition to IL-2, male patients present elevated levels of other inflammatory markers such as IL-1, IL-6, and asymmetrical dimethylarginine (ADMA, the inhibitor of NOS), as well as IgA, IgG, and IgM [248, 249]. Interestingly, chronic periodontal inflammation is considered a cause of endothelial dysfunction by inducing reactive oxygen species production in response to NO synthesis inhibition by ADMA, that in turn contributes to the development of cardiovascular diseases, which are more frequent in men than in women [248].

As it has been exposed in other sections of this review, gender prevalence in diverse bacterial infections is mainly associated to differential sex-linked immune responses regulated by sex hormones. However, host metabolic pathways, as well as expression and activity of bacterial virulence factors, are also affected by hormone action, which can influence the development of adverse sequels of infections that also show gender-associated predominance (Table 1).

## Conclusions

Sexual dimorphism in bacterial infections is attributed to socioeconomic, lifestyle, behavioral, and biological factors. The latter was reviewed in the present study and is represented by genetic factors and sex steroid hormone levels and their actions in the host, as well as by the specific bacterial species that produce infection. In general, males are more susceptible to gastrointestinal and respiratory bacterial diseases and sepsis, while females are more susceptible to urinary tract bacterial infections; however, there are conflicting data regarding other specific bacterial diseases. Sexual dimorphism is in part explained by the pro-inflammatory properties of estradiol and by the anti-inflammatory effects of testosterone via the interaction with their specific intracellular receptors. However, the participation of other hormone receptors such as PR and other membrane or intracellular receptors in the gender bias of bacterial infections has not been elucidated. Interestingly, female X chromosome mosaicism provides an advantage to females due to the expanded repertoire of immune functions. Further studies are required to clarify contradictory data and to elucidate the molecular pathways of sex steroid hormone actions involved in the gender bias of bacterial infections in order to use sex hormones, their synthesis pathways, and their receptors as possible host-directed therapeutic targets.

## Abbreviations

ADMA: Asymmetrical dimethylarginine
AR: Androgen receptor
CF: Cystic fibrosis
CLP: Cecal ligation and puncture
DHEA: Dehydroepiandrosterone
DHT: 5α-dihydrotestosterone
DMPA: Medroxyprogesterone acetate
EHEC: Enterohemorrhagic *Escherichia coli*
ER: Estrogen receptor
HIV: Human immunodeficiency virus
IBS: Irritable bowel syndrome
IFN: Interferon
Ig: Immunoglobulin
IL: Interleukin
iNOS: Inducible nitric oxide synthase
LBP: LPS-binding protein
LPS: Lipopolysaccharide
MHC: Major histocompatibility complex
NK: Natural killer
NO: Nitric oxide
PR: Progesterone receptor
TGF: Transforming growth factor
TIP: Typhoid ileal perforation
TLR: Toll-like receptor
TNF: Tumor necrosis factor
